# Adipokines and Obesity Are Associated with Colorectal Polyps in Adult Males: A Cross-Sectional Study

**DOI:** 10.1371/journal.pone.0085939

**Published:** 2014-01-17

**Authors:** Sarah S. Comstock, Kari Hortos, Bruce Kovan, Sarah McCaskey, Dorothy R. Pathak, Jenifer I. Fenton

**Affiliations:** 1 Department of Food Science and Human Nutrition, Michigan State University, East Lansing, Michigan, United States of America; 2 College of Osteopathic Medicine, Michigan State University, East Lansing, Michigan, United States of America; 3 Tri-County Gastroenterology, Professional Corporation, Clinton Township, Michigan, United States of America; 4 Department of Epidemiology and Biostatistics, Michigan State University, East Lansing, Michigan, United States of America; University of Munich, Germany

## Abstract

**Background:**

Obesity increases the risk of colon cancer. It is also known that most colorectal cancers develop from adenomatous polyps. However, the effects of obesity and adipokines on colonic polyp formation are unknown.

**Methods:**

To determine if BMI, waist circumference or adipokines are associated with colon polyps in males, 126 asymptomatic men (48–65 yr) were recruited at time of colonoscopy, and anthropometric measures as well as blood were collected. Odds ratios were determined using polytomous logistic regression for polyp number (0 or ≥3) and polyp type (no polyp, hyperplastic polyp, tubular adenoma).

**Results:**

41% of the men in our study were obese (BMI ≥30). The odds of an obese individual having ≥3 polyps was 6.5 (CI: 1.3–33.0) times greater than those of a lean (BMI<25) individual. Additionally, relative to lean individuals, obese individuals were 7.8 (CI: 2.0–30.8) times more likely to have a tubular adenoma than no polyp. As BMI category increased, participants were 2.9 (CI: 1.5–5.4) times more likely to have a tubular adenoma than no polyps. Serum leptin, IP-10 and TNF-α were significantly associated with tubular adenoma presence. Serum leptin and IP-10 were significantly associated with increased likelihood of ≥3 polyps, and TNF-α showed a trend (p = 0.09).

**Conclusions:**

Obese men are more likely to have at least three polyps and adenomas. This cross-sectional study provides evidence that colonoscopy should be recommended for obese, white males.

## Introduction

The prevalence of obesity has risen dramatically over the past 20 years in the United States and other developed countries [1], [2]. Obesity is a risk factor for many diseases, including cancer. Although a firm association exists between obesity and colorectal cancer [3]–[6], the mechanisms leading to this increased risk are unknown. Despite the fact that most colorectal cancers develop from adenomatous polyps [7], the associations between obesity and colonic polyp promotion have been only minimally studied [8]–[10].

Adipose tissue is recognized as an endocrine organ producing a variety of proteins, hormones and cytokines that are referred to collectively as adipokines [11]. These adipokines possess broad biological activities, including homeostatic and pathologic functions. Adipose tissue in general and visceral adipose tissue in particular is thought to be a key regulator of systemic inflammation [12]. As visceral adiposity increases so does the release of pro-inflammatory adipokines including IL-6, leptin and TNF-α. This is accompanied by decreased release of anti-inflammatory adipokines, including adiponectin [12]. It is hypothesized that elevated inflammatory adipokines contribute to the process of carcinogenesis [13].

The complex etiology of colon cancer makes inference between individual serum adipokine concentrations and risk of the disease controversial. Beyond the brief list of adipokines mentioned above, it is unknown what specific adipokine(s) may contribute to colon carcinogenesis. Given the difficulty in controlling multiple confounding variables in the human population, data are sparse regarding the systemic proteins that change in response to adiposity in individuals as they develop cancer. Thus, there is a lack of data supporting the association of adipokines with specific anthropomorphic patterns and colon cancer risk. To control for effects of estrogen and fat distribution, only adult males were enrolled in the study. This study provides evidence that, in addition to body mass index (BMI) and waist circumference, specific adipokines are associated with increased numbers of colorectal polyps and tubular adenoma formation in otherwise healthy adult males in a community setting.

## Materials and Methods

### Ethics Statement

The study was approved by the Biomedical and Health Institutional Review Board of Michigan State University (IRB# 08-786). The Biomedical and Health Institutional Review Board is one of three IRB committees on the Michigan State University East Lansing campus. Michigan State University's IRBs were established to advance the goal of conducting research with diligence and integrity. The purpose of the committee is to protect the rights, welfare and privacy of human subjects who participate in research conducted by students and/or faculty affiliated with MSU. At the time of enrollment, immediately prior to routine colonoscopy, written informed consent was obtained from each participant.

### Study Population

Between August 2009 and February 2011, healthy males ranging from 48–65 years of age were recruited from either Tri-County Gastroenterology Clinic (Macomb, MI) or Michigan State University Clinic (East Lansing, MI) at the time of colonoscopy. These individuals were undergoing screening colonoscopies and were asymptomatic. Exclusion criteria included: 1) cancer within the past two years, 2) surgery within the past two years, 3) inflammatory bowel diseases (e.g., Crohn's, ulcerative colitis), 4) autoimmune disorders (e.g., Rheumatoid arthritis, HIV/AIDS, Lupus), 5) diabetes, 6) chronic liver or kidney disease, 7) history of heart failure, 8) current immunosuppressant usage (e.g., Prednisone), 9) asthma, chronic obstructive pulmonary disease or other lung problems, 10) familial adenomatous polyposis, and 11) Lynch syndrome or hereditary non-polyposis colorectal cancer. 126 men (>96% white) participated in the study. At the time of enrollment, immediately prior to routine colonoscopy, written informed consent was obtained, and clinical metadata on subject co-morbidities, current medications, and family history were collected. Also at the time of enrollment, venous blood was drawn and serum was isolated by standard procedures and stored at −80°C.

#### Anthropometry

At the time of enrollment, anthropometric measures were taken to calculate BMI and to record waist circumference. Trained staff measured participants' height, weight, and waist circumference following standard procedures [14]. The staff attended two training sessions and demonstrated acceptable inter-observer reliability for all measures. All measurements were conducted in duplicate, and the average of two measures was used for analysis. Scales and stadiometers were calibrated with standard weights and heights prior to the first measurement of the day. Height was measured to the closest 0.1 cm using a wall-mounted stadiometer (SECA 214; Seca Corp, British Indicators Ltd., Birmingham, UK). Body weight was measured to the closest 0.2 kg on a digital platform scale accurate to 200 kg (BWB-800AS Digital Scale, Tanita, Arlington Heights, IL). Body weight was measured in light clothing with empty pockets and without shoes, watches, and other accessories. BMI was calculated as weight (kg) divided by height squared (m^2^). Weight status was defined as lean (BMI <25), overweight (25≤ BMI <30) and obese (BMI ≥30).

### Colonoscopy Interpretation

Full colonoscopy was performed on each participant. During the colonoscopy, a gastroenterologist (MSU-affiliated clinics, MI) categorized the segment of the colon in which polyps were found. Specimens collected during colonoscopy were sent to regional medical center Pathology Departments for histopathological analysis by board-certified pathologists.

### Serum Adipokine Analysis

Multimeric adiponectin (total, high (HMW), medium (MMW) and low (LMW) molecular weight) was measured by ELISA following the manufacturer's instructions (Alpco Diagnostics, Salem, NH). Human leptin ELISA kits were used according to the manufacturer's instructions (R&D Systems, DY398; Minneapolis, MN). The Synergy HT plate reader (Bio-Tek, Winooski, VT) was used to measure ELISA absorbances. Serum was used to assess systemic cytokine concentrations. Serum concentrations of interleukin (IL)-6, tumor-necrosis factor-α (TNF-α), and interferon-γ-inducible protein-10 (IP-10, also known as CXCL10) were determined using a MILLIPLEX™ MAP Human Cytokine/Chemokine panel (HCYTOMAG-60K, Millipore, Billerica, MA) as per the manufacturer's protocol. Samples were analyzed on a Bio-Plex 100 using Bio-Plex 4.1 software (Bio-Rad, Hercules, CA).

### Statistical Analyses

Frequencies, means, standard deviations and ranges were calculated for descriptive analysis. Correlations among variables were estimated by Pearson correlation coefficients (**Table 1**). Categorical variables were constructed using either biological cut off points (BMI, polyp number, polyp type) or tertiles within the data (waist circumference, leptin, adiponectin, HMW, MMW, LMW, IL-6, TNF-α, IP-10). For most analyses, BMI was set to three categories: <25, ≥25 but <30, or ≥30. However, in the analysis of polyp presence and type by location, BMI was analyzed as a continuous variable due to the small number of participants in each subgroup at time of cross-tabulations. Polyp number was set to four categories: 0, 1, 2, or ≥3 polyps. Polyp type was set to three categories: no polyps, hyperplastic polyp(s) or tubular adenoma(s). The presence of hyperplastic polyps puts an individual at low risk for cancer. Tubular adenomas, also called adenomatous polyps or adenomas, carry higher risk for cancer [7]. For polyp type, each individual was assigned a single score based on the colorectal polyp with the most pathology. For proximal and distal colon polyp location variables, 0 indicated no polyps present and 1 indicated ≥1 polyp present in the specified location.

**Table 1:** Pearson correlation coefficients.

			Adiponectin						
	Waist circumference	Leptin	Total	HMW	MMW	LMW	IL-6	IP-10	TNF-α
BMI	**0.8870**	**0.7437**	**−0.3881**	**−0.3306**	**−**0.1630	**−0.4011**	0.1594	**0.3137**	**0.2655**
	**<0.0001**	**<0.0001**	**<0.0001**	**0.0002**	0.0681	**<0.0001**	0.0758	**0.0004**	**0.0028**
Waist Circumference		**0.7404**	**−0.3627**	**−0.2858**	**−**0.1131	**−0.4401**	0.06340	**0.2064**	**0.2045**
		**<0.0001**	**<0.0001**	**0.0012**	0.2074	**<0.0001**	0.4784	**0.0209**	**0.0222**
Leptin			**−0.2467**	**−0.2220**	**−**0.0515	**−0.2586**	0.1106	**0.4114**	**0.3469**
			**0.0054**	**0.0125**	0.5667	**0.0035**	0.2195	**<0.0001**	**<0.0001**
Total Adiponectin				**0.9523**	**0.6217**	**0.7334**	**−**0.1135	**−**0.1606	**−0.1945**
				**<0.0001**	**<0.0001**	**<0.0001**	0.2075	0.0735	**0.0298**
HMW Adiponectin					**0.5650**	**0.5311**	**−**0.2199	**−**0.1388	**−0.1885**
					**<0.0001**	**<0.0001**	0.1488	0.1226	**0.0353**
MMW Adiponectin						**0.1917**	**−**0.0362	**−**0.0679	**−**0.1244
						**0.0316**	0.6890	0.4518	0.1670
LMW Adiponectin							**−**0.0585	**−**0.1618	**−**0.1341
							0.5168	0.0714	0.1360
IL-6								**0.3276**	**0.5684**
								**0.0002**	**<0.0001**
IP-10									**0.5175**
									**<0.0001**

Note: The top number is the Pearson correlation coefficient. The P values are shown under the correlation coefficient.

IL-6, TNF-α, and IP-10 values were missing for 1 individual (0.8% missing-ness). Age was treated as a continuous variable. Each individual was assigned a smoking status of “never smoked” or “ever smoked”. An individual was classified as “never smoked” if he had smoked fewer than 100 cigarettes over the course of his lifetime. Smoking status was missing for 22 individuals (17.5% missing-ness). All missing data were considered missing at random.

Multiple imputation (seed  = 20121119, imputations  = 7) was used to impute the 1 value missing for IL-6, TNF-α, IP-10 and all missing smoking data [15]. When testing the relationship between polyp number and polyp type with anthropometric measures of obesity, BMI and waist circumference, and with the adipokines, leptin, IL-6, TNF-α, IP-10 and adiponectin (total, HMW, MMW, LMW), all of these factors were used in the imputation algorithm of missing values, except HMW which was highly correlated with total adiponectin. When testing the relationship between proximal (right) colon polyp location with BMI, the following variables were used in the imputation: age, BMI, proximal colon polyp presence, and smoking. Polyp type was not used in the imputation because it was highly correlated with polyp presence. When testing the relationship between distal (left) colon polyp location with BMI, the following variables were used in the imputation: age, BMI, distal colon polyp presence, and smoking. Polyp type was not used in the imputation because it was highly correlated with polyp presence. When testing the relationship between the presence of a tubular adenoma in the proximal colon with BMI, the following variables were used in the imputation: age, BMI, proximal colon polyp type, and smoking. When testing the relationship between the presence of a tubular adenoma in the distal colon with BMI, the following variables were used in the imputation: age, BMI, distal colon polyp type, and smoking.

Odds ratios (OR) were determined using polytomous logistic regression models for categorical outcome data with more than two levels. Otherwise odds ratios were determined using logistic regression. All models were adjusted for age and smoking status. Test for trend was carried out across categories for the factors of interest. Because imputation was used, multiple imputation analyze (Proc MIANALYZE) was used to determine the results from analysis of the imputed data sets. SAS version 9.3 (SAS Institute Inc., Cary, NC) was used for all statistical analysis. p≤0.05 indicates significance.

## Results

Age, BMI, and waist circumference of participants are given in **Table 2**. Of the 126 men screened, 57 (45%) had at least one polyp, and 23 (18%) had ≥3 polyps. Only 4 (3.2%) participants (BMI  = 31.8, 37.0, 40.9 and 40.5) had more than 5 polyps. 37 (29.4%) of the participants had a tubular adenoma. Only three participants had advanced adenomas (one with villous histology and two with adenomas 1 cm or larger). All three of these individuals were obese. Age did not vary across these groups, however both BMI and waist circumference increased with increasing number of polyps and the presence of a tubular adenoma. In addition, 17 (13.5%) of the participants had ≥3 polyps with at least one tubular adenoma. 52 (41%) of the participants were obese, 47 (37.3%) were overweight, and 27 (21.4%) were lean.

**Table 2:** Participant† Characteristics.

	Overall	No Polyps	Any Polyps	1 Polyp	2 Polyps	≥3 Polyps	Hyperplastic Polyp	Tubular Adenoma
		n = 69	n = 57	n = 23	n = 11	n = 23	n = 20	n = 37
Age‡	57	57	57	57	57	57	57	57
(years)	(48–65)	(48–65)	(50–65)	(51–65)	(52–65)	(50–65)	(50–65)	(50–65)
BMI‡	29.7	28.4	31.3	30.6	30.3	32.5	29.5	32.3
(kg/m^2^)	(19.2–45.6)	(21.7–39.1)	(19.2–45.6)	(22.0–42.8)	(20.6–42.9)	(19.2–45.6)	(22.0–40.9)	(19.2–45.6)
Waist circumference‡	41.4	40.1	42.9	42.1	43.1	43.7	41.6	43.6
(inches)	(29.8–57.5)	(30.0–55.0)	(29.8–57.5)	(29.8–54.5)	(31.0–52.0)	(31.8–57.5)	(34.0–52.0)	(29.8–57.5)

All participants (n = 126) were male, >96% Caucasian

Reported as mean (range)

**Figure 1:**
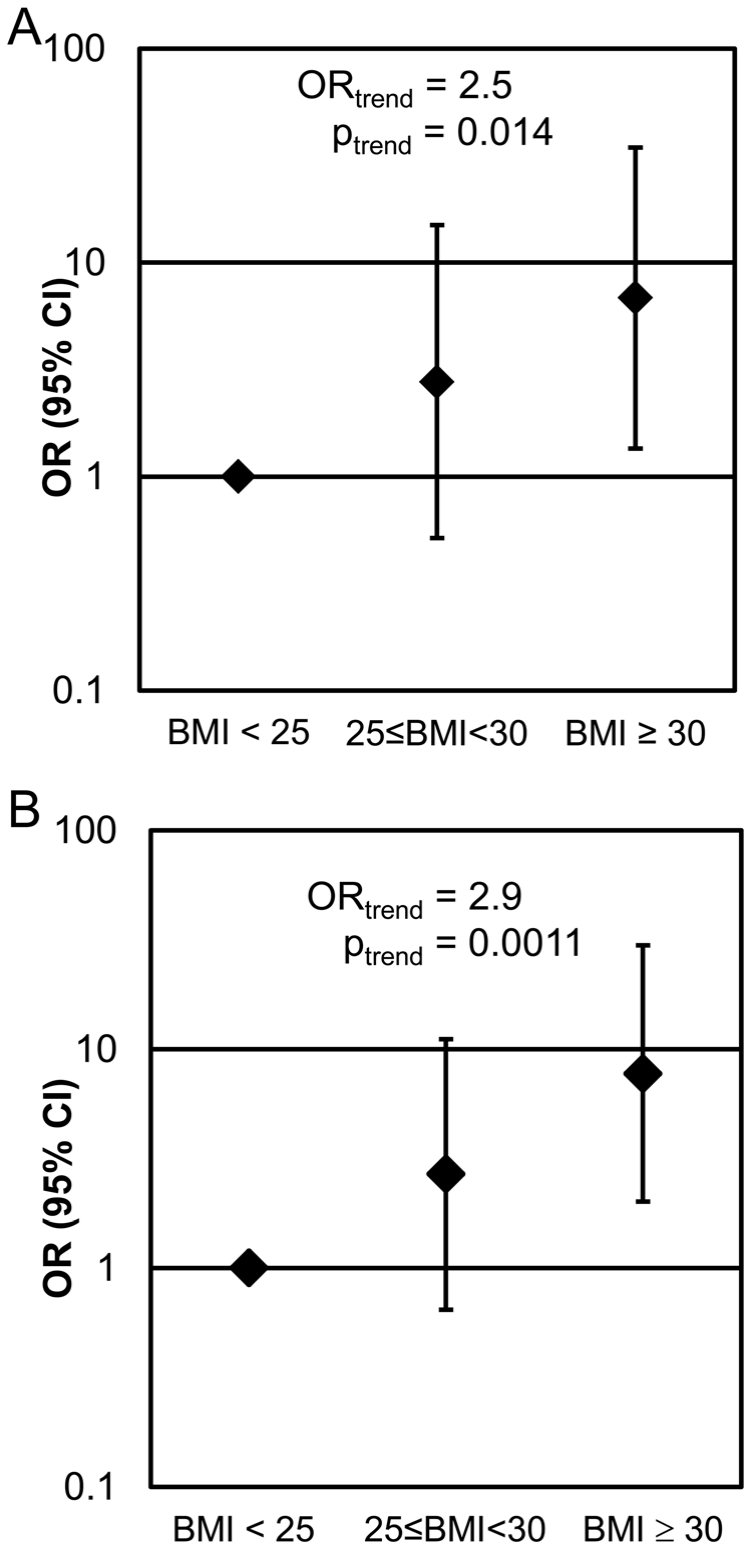
BMI is associated with polyp number (A) as well as the presence of tubular adenoma (B). A, Compared to lean males, obese males (BMI ≥30) are 6.5 times more likely to have ≥3 polyps. In addition, for each category increase in BMI, a man is 2.5 times more likely to have ≥3 polyps than no polyps. B, Compared to lean males, obese males are 7.8 times more likely to have a tubular adenoma. For each category increase in BMI, a man is 2.9 times more likely to have a tubular adenoma. The model was adjusted for age and smoking status (ever/never).

The odds that an obese participant would have ≥3 polyps were 6.5 (CI: 1.3–33.0) times greater than those that a lean participant would have ≥3 polyps (p = 0.0241, **Figure 1A**). Additionally for each category increase in BMI, a man was 2.5 (CI: 1.2–5.1) times more likely to have ≥3 polyps than no polyps (p = 0.0143). An obese participant was also more likely to have a tubular adenoma, compared to the likelihood that his lean counterpart would have a tubular adenoma (OR = 7.8, CI: 2.0–30.8; **Figure 1B**). For each category increase in BMI, a man was 2.9 times more likely to have a tubular adenoma than no polyps (p = 0.0011).

In addition to obesity being related to the overall number and type of polyps, BMI was also associated with the location of polyps. Compared to a lean individual, an obese individual was 1.2 times more likely to have a polyp in his proximal (right) colon (p = 0.0017) as well as 1.2 times more likely to have a tubular adenoma in his proximal colon (p = 0.0005, **Table 3**). Similarly, an obese individual was 1.1 times more likely to have a polyp in his distal (left) colon (p = 0.0096) as well as 1.1 times more likely to have a tubular adenoma in his distal colon (p = 0.0084, **Table 3**). 50 participants had a polyp in their distal colon (descending colon, sigmoid colon, and rectum). 24 participants had a polyp in their proximal colon (transverse colon, cecum and ascending colon). Nine of those individuals had a polyp in their ascending colon. 19 of the 24 individuals with proximal colon polyps had at least one tubular adenoma. These tubular adenomas were found in 14 obese participants, 4 overweight participants and 1 lean participant. Of the 9 individuals with ascending colon polyps, 8 had at least one tubular adenoma. Six of these individuals were obese, 1 was overweight and 1 was lean. Thus, right-sided colon polyps were more numerous in obese participants than lean individuals. In addition, obese participants were more likely to have a right-sided adenoma than lean participants. Overall, obese participants were more likely to have either a proximal or distal colon polyp than lean participants.

**Table 3:** Association of BMI with the presence or severity of polyps in the proximal† or distal†† colon.

	Proximal Colon		Distal Colon	
	OR (95% CI)	p value	OR (95% CI)	p value
Presence	1.2 (1.1–1.3)	0.0017	1.1 (1.02–1.2)	0.0096
Severity	1.2 (1.1–1.3)	0.0005	1.1 (1.03–1.2)	0.0084

Note: Model is adjusted for age and ever/never smoked.

Proximal (right) colon included: cecum, ascending colon, and transverse colon

Distal (left) colon included: descending colon, sigmoid colon, and rectum

**Figure 2:**
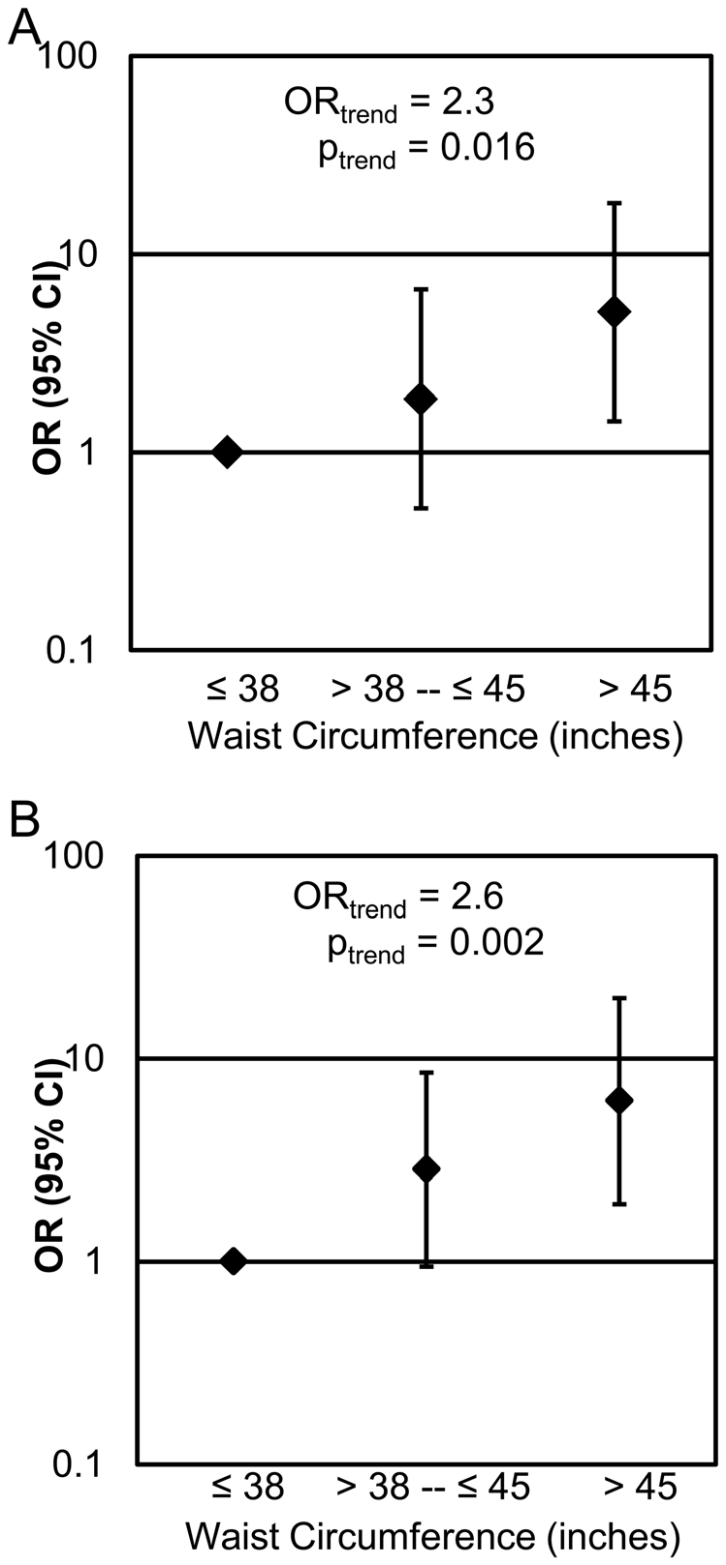
Waist circumference is associated with polyp number (A) as well as the presence of tubular adenoma (B). A, Compared to males with a waist circumference ≤38 inches, males with a waist circumference >45 inches are 4.6 times more likely to have ≥3 polyps. In addition, for each increase in waist circumference tertile, a man is 2.3 times more likely to have ≥3 polyps than no polyps. B, Compared to males with a waist circumference ≤38 inches, males with a waist circumference >45 inches are 6.2 times more likely to have a tubular adenoma. For each category increase in waist circumference tertile, a man is 2.6 times more likely to have a tubular adenoma than no adenoma. The model was adjusted for age and smoking status (ever/never).

Waist circumference was also associated with polyp number and the presence of tubular adenomas in our study population. Males with a waist circumference in the highest tertile (>45 inches) were 4.6 (CI: 1.3–16.8) times more likely to have ≥3 polyps than males with a waist circumference in the lowest tertile (≤38 inches) (p = 0.0211, **Figure 2A**). For each category increase in waist circumference, a man was 2.3 (CI: 1.2–4.6) times more likely to have ≥3 polyps than no polyps (p = 0.0163). Waist circumference was also associated with polyp type. Males with a waist circumference in the highest tertile were 6.2 (CI: 1.9–19.9) times more likely to have a tubular adenoma than those in the lowest tertile (p = 0.0023, **Figure 2B**). For each increase in waist circumference tertile, a male was 2.6 (CI: 1.4–4.6) times more likely to have a tubular adenoma (p = 0.002).

Serum adipokines including adiponectin (total, HMW, MMW, LMW), leptin, IL-6, TNF-α, and IP-10 (CXCL10) were measured. Significant associations were observed between two of these serum adipokines (leptin and IP-10) with polyp number, and a third serum adipokine (TNF-α) showed a trend towards significance (p = 0.09) (**Table 4**). Participants with the highest concentrations of serum leptin, were 3.6 (CI: 1.1–12.4) times more likely to have ≥3 polyps than those with serum leptin levels in the lowest tertile (p = 0.0378). For each increase in leptin tertile, a participant was 2.0 (CI: 1.1–3.9) times more likely to have ≥3 polyps (p = 0.0283). Participants with the highest concentrations of serum IP-10 were 4.1 (CI: 1.2–14.1) times more likely to have ≥3 polyps than those with serum IP-10 levels in the lowest tertile (p = 0.0258). For each increase in IP-10 tertile, a participant was 2.2 (CI: 1.1–4.1) times more likely to have ≥3 polyps (p = 0.0193). None of the serum adipokines were associated with the presence of hyperplastic polyps (**Table 5**). Three of these serum adipokines were associated with tubular adenomas (**Table 6**). Two adipokines (leptin and IP-10) were the same as for polyp number, and additionally, TNF-α had a statistically significant association with the presence of a tubular adenoma. Participants with the highest concentrations of serum leptin were 3.8 (CI: 1.3–11.2) times more likely to have a tubular adenoma than those with serum leptin levels in the lowest tertile (p = 0.0134). For each increase in leptin tertile, a participant was 2.0 (CI: 1.2–3.4) times more likely to have a tubular adenoma (p = 0.0115). Participants with the highest concentrations of serum IP-10 were 3.2 (CI: 1.1–8.9) times more likely to have a tubular adenoma than those with serum IP-10 levels in the lowest tertile (p = 0.0268). For each increase in IP-10 tertile, a participant was 1.8 (CI: 1.1–3.1) times more likely to have a tubular adenoma (p = 0.0223). Participants with the highest concentrations of serum TNF-α were 4.9 (CI: 1.6–14.8) times more likely to have a tubular adenoma than those with serum TNF-α levels in the lowest tertile (p = 0.0054). For each increase in TNF-α tertile, a participant was 2.4 (CI: 1.4–4.0) times more likely to have a tubular adenoma (p = 0.0055).

**Table 4:** Association of serum adipokine tertiles with having ≥3 polyps.

		Trend
		OR
	OR (95% CI)	(p trend)
Leptin (ng/ml)		
≤4.5	1	**2.0**
>4.5 to ≤9	1.4 (0.4–5.2)	**(0.0283)**
>9	**3.6 (1.1**–**12.4)**	
Total Adiponectin (µg/ml)		
≤3.6	1	1.0
>3.6 to ≤5.3	1.1 (0.3–3.8)	(0.9043)
>5.3	1.1 (0.3–3.5)	
LMW Adiponectin (µg/ml)		
≤1.6	1	0.9
>1.6 to ≤2.1	0.6 (0.2–1.8)	(0.8515)
>2.1	0.9 (0.3–2.8)	
MMW Adiponectin (µg/ml)		
≤0.5	1	0.9
>0.5 to ≤0.8	0.5 (0.2–1.9)	(0.8197)
>0.8	0.8 (0.3–2.7)	
HMW Adiponectin (µg/ml)		
≤1.2	1	1.3
>1.2 to ≤2.4	1.3 (0.4–4.5)	(0.3821)
>2.4	1.7 (0.5–5.8)	
IP-10 (pg/ml)		
≤255.3	1	**2.2**
>255.3 to ≤362.6	1.1 (0.3–4.3)	**(0.0193)**
>362.6	**4.1 (1.2**–**14.1)**	
TNF-α (pg/ml)		
≤5.9	1	1.7
>5.9 to ≤8.6	4.3 (1.2–15.8)	(0.0981)
>8.6	3.2 (0.8–12.5)	

Note: Model is adjusted for age and ever/never smoked.

**Table 5:** Association of serum adipokine tertiles with the presence of a hyperplastic polyp.

	OR (95% CI)	p trend
Leptin (ng/ml)		
≤4.5	1	0.3782
>4.5 to ≤9	2.2 (0.6–7.7)	
>9	1.8 (0.5–6.7)	
Total Adiponectin (µg/ml)		
≤3.6	1	0.7178
>3.6 to ≤5.3	1.0 (0.3–3.3)	
>5.3	0.8 (0.2–2.7)	
LMW Adiponectin (µg/ml)		
≤1.6	1	0.2128
>1.6 to ≤2.1	0.3 (0.1–0.9)	
>2.1	0.5 (0.1–1.6)	
MMW Adiponectin (µg/ml)		
≤0.5	1	0.6174
>0.5 to ≤0.8	0.5 (0.1–1.8)	
>0.8	0.7 (0.2–2.4)	
HMW Adiponectin (µg/ml)		
≤1.2	1	0.7933
>1.2 to ≤2.4	0.8 (0.2–2.5)	
>2.4	0.9 (0.3–2.9)	
IP-10 (pg/ml)		
≤255.3	1	0.7590
>255.3 to ≤362.6	1.1 (0.3–3.5)	
>362.6	1.2 (0.3–4.3)	
TNF-α (pg/ml)		
≤5.9	1	0.6428
>5.9 to ≤8.6	1.5 (0.4–4.9)	
>8.6	1.3 (0.4–4.5)	

Note: Model is adjusted for age and ever/never smoked.

**Table 6:** Association of serum adipokine tertiles with the presence of a tubular adenoma.

		Trend
		OR
	OR (95% CI)	(p trend)
Leptin (ng/ml)		
≤4.5	1	**2.0**
>4.5 to ≤9	2.1 (0.7–6.2)	**(0.0115)**
>9	**3.8 (1.3**–**11.2)**	
Total Adiponectin (µg/ml)		
≤3.6	1	1.1
>3.6 to ≤5.3	0.9 (0.3–2.5)	(0.8230)
>5.3	1.1 (0.4–3.0)	
LMW Adiponectin (µg/ml)		
≤1.6	1	0.9
>1.6 to ≤2.1	0.5 (0.2–1.3)	(0.6849)
>2.1	0.8 (0.3–2.2)	
MMW Adiponectin (µg/ml)		
≤0.5	1	0.9
>0.5 to ≤0.8	0.6 (0.2–1.8)	(0.6569)
>0.8	0.8 (0.3–2.2)	
HMW Adiponectin (µg/ml)		
≤1.2	1	1.3
>1.2 to ≤2.4	1.2 (0.4–3.3)	(0.2795)
>2.4	1.7 (0.6–4.8)	
IP-10 (pg/ml)		
≤255.3	1	**1.8**
>255.3 to ≤362.6	1.1 (0.4–3.2)	**(0.0223)**
>362.6	**3.2 (1.1**–**8.9)**	
TNF-α (pg/ml)		
≤5.9	1	**2.4**
>5.9 to ≤8.6	**3.3 (1.1–10.4)**	**(0.0055)**
>8.6	**4.9 (1.6**–**14.8)**	

Note: Model is adjusted for age and ever/never smoked.

## Discussion

Associations between BMI, waist circumference and systemic adipokines with polyp number or polyp type occur. BMI, waist circumference, leptin, and IP-10 were positively associated with the presence of ≥3 polyps. These factors as well as TNF-α were also positively associated with the presence of a tubular adenoma. BMI was positively associated with the presence of polyps and the presence of tubular adenomas in the proximal colon. Because polyps are considered precursors to colon cancer [7], these associations indicate that obesity and several obesity-associated adipokines increase the risk of colorectal cancer in adult, white males.

Our study is unique for several reasons. Although some research corroborates our observation that BMI and colorectal polyp formation are associated [8]–[10], a minority of this research is applicable to white males in the United States. In three meta-analyses [8]–[10], fewer than 43% of the included studies were conducted in the United States and none of those studies were conducted in asymptomatic, adult, white males. The majority of this research was conducted in Europe or in Asian countries, such as Japan and Korea, where individuals are typically leaner. The BMI distribution of the study population had a mean of 29.73 and a median of 29.12 (Interquartile Range  = 25.95 to 32.88). When compared to the World Health Organization (WHO) numbers for males in the United States [16], the study population is slightly heavier than average. The WHO database reports that 33% of males in the United States are normal weight, 40% overweight and 33% obese. Our study population is 21% normal weight, 37% overweight and 41% obese. With regard to public health, the results from this study are more generalizable to adult men in the United States.

Although others have reported associations between BMI and polyp formation [8]–[10], data associating adipokines with colorectal polyp formation [17]–[23] or recurrence [24] are sparse. Furthermore, few studies simultaneously evaluated several serum adipokines, and we could find no other studies evaluating the relationship between serum IP-10/CXCL10 and colorectal adenoma. Additionally, of the data that exists in this area, some was collected in populations of relatively lean individuals, such as the Japanese [23]. The current study extends these observations by associating serum concentrations of specific adipokines with polyp number and type. Since these data are observational, we cannot impute risk of colorectal adenoma presence to specific adipokines. However, these data can be discussed in light of other data that links these patterns to specific disease risk and may suggest potential criteria for improved screening recommendations.

BMI, waist circumference and some adipokines were associated with the presence of colorectal adenomas, but they were not associated with the presence of hyperplastic polyps. This is similar to at least one other report [25]. However, there are also reports of associations between some of these factors and the presence of hyperplastic polyps [26]–[28]. Hyperplastic polyps are commonly regarded as benign [29], [30], and the necessity for early identification of these polyps is controversial. Under some circumstances, hyperplastic polyps have malignant potential [31], [32]. Proper identification of hyperplastic polyps with malignant potential is difficult without molecular testing [33]. We sought to identify factors associated with polyp risk. Thus, while the inclusion of hyperplastic polyps in our analysis may over-estimate colon cancer risk, it is a true reflection of polyp risk.

We did not find an association between IL-6 concentrations or adiponectin concentrations and risk of polyp number or type. Because elevated IL-6 has been characterized as a pro-inflammatory cytokine, we expected its concentrations to be associated with polyp number and type. IL-6 was also not associated with colorectal cancer in other studies [34], [35]. However, it was associated with the presence of a colorectal adenoma [36], [37]. Adiponectin is an adipocyte-derived hormone with diverse biological functions, including regulation of metabolic pathways in adipocytes, muscle cells and peripheral tissues. It circulates as bioactive multimers, including total, HMW, MMW and LMW [38], and these multimeric forms are purported to have varying effects on cancer risk [39]. Adiponectin is emerging as a key regulator of the balance between adipose tissue and inflammation [40]. Serum levels of adiponectin exceed 10 µg/ml in normal-weight individuals [41]. However, as adiposity increases, adiponectin levels decrease, and this decrease is thought to mediate insulin resistance which may provide a role for adiponectin in colon carcinogenesis [42]. Consistent with other data from obese humans, serum adiponectin (total, HMW, LMW) was correlated with BMI and waist circumference (**Table 1**) in the study population. Thus, we expected adiponectin concentrations to be inversely associated with polyp number and type. Others have shown a significant negative association of the highest adiponectin (both total (≥5.27 µg/ml) and HMW (≥1.92 µg/ml)) concentrations with colorectal adenoma formation in adult Japanese men [23]. Also, plasma adiponectin was inversely correlated with risk of colorectal cancer in American men [43] and Japanese individuals [22]. In this study, we observed no association with IL-6 or either HMW or total adiponectin and polyp number or presence of adenoma.

Of the adipokines associated with polyp number or type, including IP-10, TNF-α and leptin, all are considered pro-inflammatory at elevated concentrations and increase the activity of signaling pathways in their target cells. They increase the phospho-inositol-3-kinase/Akt signaling pathway [44]–[46]. Increases in this signaling pathway are associated with cell survival, growth and division (reviewed in [47]) especially in the colon (reviewed in [48]). Signaling pathways such as nuclear factor-κB (NF-κB) and others are also important in cancer initiation and progression [49], [50]. Therefore, elevated inflammatory adipokines may support an inflammatory microenvironment leading to tissue damage, genetic mutation and an imbalance between apoptosis and proliferation [13].

IP-10, also known as CXCL10, is expressed in a variety of tissues including liver, gut, spleen, kidney and thymus, in response to interferon gamma. IP-10 acts as a chemoattractant for monocytes, NK cells and certain T-cells. The recruitment of these lymphocytes exacerbates inflammation and can damage tissue at the site [51]. Mature human adipocytes secrete IP-10 [52]. IP-10 is correlated with BMI in adolescents [53]. In our study population, serum IP-10 and BMI were significantly correlated (**Table 1**). However, in a study comparing Japanese men in Japan, and white and Japanese men in the United States, IP-10 and BMI were only significantly correlated in Japanese men in the United States (r^2^ = 0.407, p<0.01). High serum concentrations of IP-10 are associated with poorer prognosis for colorectal cancer (CRC) patients [54]. However, high tumor IP-10 mRNA expression was associated with better prognosis [55], [56]. In fact, when tumors were treated with IP-10, isolated infiltrating lymphocytes were better killers and produced higher levels of IFN-γ [57]. It may be that IP-10 in the serum is indicative of a systemic immune response to polyps in the colon. Thus, serum IP-10 (>362.6 pg/ml) may be an effective biomarker for colorectal polyps.

The quintessential cancer-associated cytokine, TNF-α [58] was strongly associated with the presence of a tubular adenoma but not with polyp number in our study population. No association of tubular adenoma presence with TNF-α concentrations (highest tertile >2.98 pg/ml) was reported for adult Japanese men [23]. TNF-α was not associated with CRC in other studies [34]. In a recent study analyzing the association between plasma cytokines and the presence of colorectal adenoma, TNF-α was higher in individuals with colorectal adenomas than in controls [59]. Thus, TNF-α (>5.9 pg/ml) may indicate the presence of adenomas with more advanced pathology.

Leptin was strongly associated with number and type of colorectal polyps in this study population. Consistent with other data from obese humans [60], serum leptin was correlated with BMI and waist circumference (**Table 1**). Leptin belongs to the class I cytokine family and possesses immunomodulatory activities [61], [62]. An association between serum leptin concentration and colorectal adenoma has been previously reported [20], [23]. High serum leptin was also correlated with a decreased risk of recurrent adenoma [24]. It is of interest that even obese mice demonstrated a significant inhibition of colorectal tumor cell growth when they were genetically-derived to be deficient in either leptin or leptin-receptor [63].

The mechanism by which BMI and body size influence colorectal polyp formation and cancer risk is not understood. However, evidence from studies conducted in cell lines indicates that the associations we have observed are likely mechanistically relevant. Specifically, adipokines produced by adipose tissue exert an influence on the six hallmarks of cancer [64], [65]. Treatment of several conditionally immortal epithelial cell lines with leptin induced angiogenesis [66], proliferation [67]–[69] and epithelial cell production of IL-6 [68] (reviewed in [65]). A wide variety of immune, normal epithelial and epithelial-derived tumor cell types including colon epithelial cells express the leptin receptor, which mediates leptin's biological effects [46], [70]. Thus, adipokines may mediate cellular conversations between adipose, immune and epithelial cells leading to polyp formation and ultimately cancer.

This study had several strengths. The design of this study, cross-sectional with consecutive enrollment, reduced enrollment bias. Additionally, only asymptomatic individuals undergoing a complete screening were enrolled. Anthropometric measurements were taken by trained staff. Participants provided information about relevant confounding variables, thus allowing for control of these confounding variables in our analysis. Our population is fairly defined, and the results are likely typical for men of an ethnicity and age similar to that of the study population.

Caution must be used when interpreting the results of this study as there are some limitations. Because this study was conducted in primarily white, adult males, the results may not apply to individuals of other ethnicities or females. Although, individuals with diagnosed pre-diabetes/diabetes or taking medications related to glucose dysregulation were excluded from the study, it is possible some participants had hyperglycemia and/or hyperinsulinemia. Exclusion of individuals with known diabetes, a confounder for colon cancer development, decreased the required enrollment size. Some participants had prior colonoscopies and thus may have had previously identified polyps. Our smoking classification of “ever” or “never” does not reflect the extent of smoking. In addition, smoking classification was imputed for 22 participants. Thus, these factors may confound our findings. The associations described in this study were based on cross-sectional data thus cause cannot be assigned to any factors identified as associated with polyp number or presence of adenoma. In addition, local production rather than serum concentrations of adipokines may be more relevant to colorectal polyp formation. However, local production is not easily measured, and thus serum measurements are more useful in a clinical setting. A larger sample size would decrease the width of the reported 95% confidence intervals. Thus, this research should be repeated with a larger sample size.

Despite these caveats and because it is known that removal of polyps decreases the incidence of colon cancer [7], this research provides additional evidence that colonoscopy should be recommended for obese, white males. Furthermore, this research suggests that high serum concentrations of leptin (>9 ng/ml), IP-10 (>362.6 pg/ml) and TNF-α (>5.9 pg/ml) could also be used to identify individuals at increased risk for colorectal polyp formation and presence of adenoma. Colonoscopy screening of individuals with these risk factors may decrease colorectal cancer rates. Studies evaluating the effectiveness of early screening programs based on these risk factors are needed.
